# β3-adrenergic receptor gene, body mass index, bone mineral density and fracture risk in elderly men and women: the Dubbo Osteoporosis Epidemiology Study (DOES)

**DOI:** 10.1186/1471-2350-7-57

**Published:** 2006-07-05

**Authors:** Claire Y Wang, Nguyen D Nguyen, Nigel A Morrison, John A Eisman, Jacqueline R Center, Tuan V Nguyen

**Affiliations:** 1 Bone and Mineral Research Program, Garvan Institute of Medical Research, St Vincent's Hospital, University of New South Wales, Sydney, NSW, Australia; 2 Faculty of Medicine, University of New South Wales, Sydney, NSW, Australia; 3 School of Medical Science, Griffith University, QLD, Australia

## Abstract

**Background:**

Recent studies have suggested that the *Arg *allele of β3-adrenergic receptor (ADRB3) gene is associated with body mass index (BMI), which is an important predictor of bone mineral density (BMD) and fracture risk. However, whether the ADRB3 gene polymorphism is associated with fracture risk has not been investigated. The aim of study was to examine the inter-relationships between ADRB3 gene polymorphisms, BMI, BMD and fracture risk in elderly Caucasians.

**Methods:**

Genotypes of the ADRB3 gene were determined in 265 men and 446 women aged 60+ in 1989 at entry into the study, whose BMD were measured by DXA (GE Lunar, WI USA) at baseline. During the follow-up period (between 1989 and 2004), fractures were ascertained by reviewing radiography reports and personal interviews.

**Results:**

The allelic frequencies of the *Trp *and the *Arg *alleles were 0.925 and 0.075 respectively, and the relative frequencies of genotypes *Trp/Trp*, *Trp/Arg *and *Arg/Arg *0.857, 0.138 and 0.006 respectively. There was no significant association between BMI and ADRB3 genotypes (p = 0.10 in women and p = 0.68 in men). There was also no significant association between ADRB3 genotypes and lumbar spine or femoral neck BMD in either men and women. Furthermore, there were no significant association between ADRB3 genotypes and fracture risk in both women and men, either before or after adjusting for and, BMD and BMI.

**Conclusion:**

The present data suggested that in Caucasian population the contribution of ADRB3 genotypes to the prediction of BMI, BMD and fracture risk is limited.

## Background

Osteoporosis and obesity are two common disorders that affect a large number of elderly in the general population [1-3]. Approximately 30% of women and 12% of men are affected by osteoporosis or low bone mass at some point during life [4]. Moreover, about 31% of elderly men and 35% of elderly women are classified as having obesity in the US [5]. Several lines of epidemiologic evidence suggest that the two disorders may be inversely associated, with obese individuals having higher bone mineral density (BMD) and reduced risk of fracture than non-obese individuals [6-11]. Indeed, it has been well-known that between 23% and 47% variance of BMD in the general population can be "explained" by the variation in body mass index (BMI) [12,13], making BMI one of the most robust and consistent predictors of BMD [14]. Both body weight (or BMI) and BMD are partly genetically determined. Twin studies have suggested that genetic factors may account for up to 80% of the BMD variance [15-17]. Likewise, between 43 to 70% of BMI variance is attributable to genetic factors [18,19]. Under the hypothesis of shared genetic effects, a gene that is associated with BMI may also protect against osteoporosis.

The variation in BMD is mainly attributable to genetic factors [16,17,20] and the liability to osteoporotic fracture is also partly determined by genetic factor [21-23]. Experimental evidence suggested that β3-adrenergic stimulation can induce expression of osteoclast differentiation factor in osteoblast cells, leading to a stimulation of osteoclastogenesis [24]. Moreover, the activation of β3-adrenergic receptors on two osteoblast-like cells can cause bone resorption in intact mouse calvariae [25]. In epidemiological studies, the β3-adrenergic receptor (ADRB3) polymorphisms have been found to be associated with BMI [26] and BMD [27]. However, such associations, while largely found in the Japanese population, have not been well studied in Caucasian populations. Moreover, the association between ADRB3 polymorphisms and fracture risk, the ultimate outcome of osteoporosis has not been reported. The present study was aimed at examining the inter-relationships between ADRB3 gene polymorphisms, body mass index, bone mineral density and fracture risk in a well-defined sample of elderly Caucasians.

## Methods

### Study design and setting

The present study was part of the Dubbo Osteoporosis Epidemiology Study (DOES), of which the details of study design and protocols have been described previously [28-30]. Briefly, DOES is a community-based, epidemiological study, where participants were recruited from the Dubbo city, a semi-urban city 400 km northwest of Sydney, Australia, with a population of approximately 32 000 people, 98.6% Caucasian, of whom 1 581 men and 2 095 women were aged 60 years or above in 1989. Dubbo's relative isolation in terms of medical care allows virtually complete ascertainment of all fractures. From 1993, blood samples were collected from individuals who volunteer to donate at initial visits or follow-up visits. Our present study reports data on a random sample of 446 women and 265 men aged 60 years or above in 1989, who had consented to donate blood for DNA analysis.

### Measurements

After obtaining written informed consent, participants were interviewed by a trained research nurse at initial and subsequent visits at approximately 2-year intervals. A structured questionnaire was used to collect data including age, anthropometric variables, life style and clinical data [31]. Bone mineral density (g/cm^2^) was measured at the lumbar spine and femoral neck by dual-energy X-ray absorptiometry (DXA) using a Lunar DPX-L densitometer (GE Lunar, WI. USA). The measurement was performed at the same visit or within one month of the blood sample collection in 99% of cases. In the cases in which the blood was taken at a time other than the scheduled visit, the BMD used was that closest to the time of blood collection.

### Ascertainment of fracture

The incidence of fracture was ascertained during the study period (1989–2004) by radiologist's report and confirmed by personal interview. Only low trauma fractures were included (i.e. a fall from the standing height or less). Fractures due to motor vehicle accidents or pathological conditions were excluded. Fractures were categorised into 4 groups: hip, symptomatic vertebral, Colles' and other fractures.

### Genotyping

Genomic DNA was extracted from peripheral blood leukocytes. The ADRB3 polymorphism was determined by polymerase chain reaction (PCR) performed with 20 ng of genomic DNA with upstream primer 5' CCA GTG GGC TGC CGA GGG 3' and downstream primer 5' GCC AGT GGC GCC CAA CGG 3' [32]. The amplification products were digested with the restriction enzyme *Bst*OI (Promega, Madison, WI, USA) and genotyped as *Trp *homozygotes (*Trp*/*Trp*), heterozygotes (*Trp*/*Arg*), or *Arg *homozygotes (*Arg*/*Arg*). This *Trp*64*Arg *polymorphism has been commonly used in genetic epidemiological studies.

### Statistical analysis

Alleles were counted and the genotype frequencies of the polymorphism was checked for Hardy-Weinberg equilibrium (HWE) by the likelihood Chi-square statistic [33]. The baseline characteristics of the subjects in the fracture group and the non-fracture group were compared using descriptive statistical tests with appropriate techniques (e.g. Student's t-test for normally distributed variables, such as BMI and BMD) and Chi-square (for categorical data), depends on the distribution of the variables. The association between each polymorphism and BMD or BMI was analyzed by the analysis of covariance (ANCOVA) model taking into account the effects of potential covariates such as age, weight and life-style factors.

In order to quantify the risk of sustaining a fracture in relation to ADRB3 genotypes, subjects were grouped into those with *Arg *allele and those without *Arg *allele. The association was analyzed by the logistic regression model taking into account the effects of potential confounders.

In order to determine the association between BMI and ADRB3 polymorphisms, a Bayesian approach was used [34,35]. In the Bayesian analysis, the currently observed data are combined with known data to derive a "posterior distribution" with credible interval. In the present study, a prior mean difference (M_0_) in BMI between subjects with and without *Arg *allele was obtained from a meta-analysis [26] with a 95% confidence interval (95%CI) being (L_0_, U_0_), from which the standard deviation (SD_0_) for the prior distribution was calculated as follow: SD_0 _= (U_0_- L_0_)/3.92, assuming the difference follows the normal distribution. In order to construct a posterior distribution with mean (M_p_) and standard deviation (SD_p_), the prior distribution was combined with the present study's data, which has a mean of M_d _and standard deviation of SD_d_. The posterior mean and SD are: M_p _=(SD_p_)^2 ^[(M_0_/SD_0_^2^)+(M_d_/SD_d _^2^)] and SD_*p *_= 1/(1/SD0)2+(1/SDd)2
 MathType@MTEF@5@5@+=feaafiart1ev1aaatCvAUfKttLearuWrP9MDH5MBPbIqV92AaeXatLxBI9gBaebbnrfifHhDYfgasaacH8akY=wiFfYdH8Gipec8Eeeu0xXdbba9frFj0=OqFfea0dXdd9vqai=hGuQ8kuc9pgc9s8qqaq=dirpe0xb9q8qiLsFr0=vr0=vr0dc8meaabaqaciaacaGaaeqabaqabeGadaaakeaadaGcaaqaaiabcIcaOiabigdaXiabc+caViabdofatjabdseaenaaBaaaleaacqaIWaamaeqaaOGaeiykaKYaaWbaaSqabeaacqaIYaGmaaGccqGHRaWkcqGGOaakcqaIXaqmcqGGVaWlcqWGtbWucqWGebardaWgaaWcbaGaemizaqgabeaakiabcMcaPmaaCaaaleqabaGaeGOmaidaaaqabaaaaa@3E21@. Thus, the posterior mean is a weighted average of two estimates (prior mean and the present study's mean) with the weight being the inverse variances. The analysis was conducted via the SAS system [36].

## Results

### Characteristics of study subjects

In total, 446 women and 265 men who had been followed-up for the median of 13.2 years (interquartile range, IQR: 10.4–14.1) and 12.9 years (IQR: 9.0–14.0) for women and men, respectively.

The frequencies of the *Trp*/*Trp*, *Trp*/*Arg *and *Arg*/*Arg *genotypes were 385 (86.3%), 58 (13.0%) and 3 (0.7%) respectively for women, and 224 (84.5), 40 (15.1%) and 1 (0.4%) respectively for men. In both sexes, the allelic frequencies of *Trp *allele and *Arg *allele were 0.925 and 0.075 respectively. Because the frequency of *Arg *allele was low in the population, individuals with *Trp*/*Arg *and *Arg*/*Arg *genotypes were considered as one group. The overall genotype distribution in the study subjects was in Hardy-Weinberg equilibrium (χ^2 ^= 0.007, p = 0.98).

Clinical characteristics of subjects were stratified by the presence or absence of *Arg *allele (Table 1). The presence of *Arg *allele in women is associated with a 0.25 SD increase in body weight, but this difference was not statistically significant (p = 0.08). In both women and men, there were no statistically significant differences between *Arg *allele carriers and non-carriers in terms of anthropometric characteristics and lifestyle factors.

**Table 1:** Baseline characteristics of subjects stratified by ADRB3 genotype

	*Trp*/*Trp*	*Trp*/*Arg *+ *Arg*/*Arg*	Standardized difference	P value
**Women (N)**	385	61		
Age (y)	71 ± 8	71 ± 6	0.00	0.47
Weight (kg)	64 ± 12	67 ± 13	0.25	0.08
Height (cm)	160 ± 6	160 ± 6	0.00	0.90
BMI (kg/m^2^)	25 ± 5	26 ± 5	0.20	0.10
Ca intake (mg/day)	644 ± 372	650 ± 355	0.02	0.90
Physical activity (METs)	80 ± 30	82 ± 23	0.07	0.54
Femoral neck BMD (g/cm^2^)	0.76 ± 0.13	0.79 ± 0.13	0.23	0.24
Lumbar spine BMD (g/cm^2^)	1.00 ± 0.18	1.04 ± 0.21	0.22	0.11
Age of menopause (y)	47 ± 7	46 ± 7	-0.14	0.52
Current/ex smoker ^a,b^	106 (27.5)	22 (36.1)		1.87

**Men (N)**	224	41		
Age (y)	70 ± 6	68 ± 5	-0.34	0.12
Weight (kg)	78 ± 12	77 ± 13	-0.08	0.46
Height (cm)	174 ± 6	173 ± 8	-0.16	0.77
BMI (kg/m^2^)	26 ± 3	26 ± 4	0.00	0.68
Ca intake (mg/day)	657 ± 367	732 ± 317	0.21	0.23
Physical activity (METs)	76 ± 49	72 ± 48	-0.08	0.69
Femoral neck BMD (g/cm^2^)	0.92 ± 0.15	0.93 ± 0.15	0.07	0.63
Lumbar spine BMD (g/cm^2^)	1.26 ± 0.21	1.24 ± 0.24	-0.09	0.59
Current/ex smoker ^a,b^	122 (54.5)	25 (61.0)		0.59

Values are mean ± SD, otherwise specified; P values were calculated using unpaired t-test, otherwise specified. BMI, body mass index; Ca, calcium; METs, metabolic equivalents.

^a ^N(%); ^b^Chi-square test.

### ADRB3 genotypes and BMI

**Figure 1:**
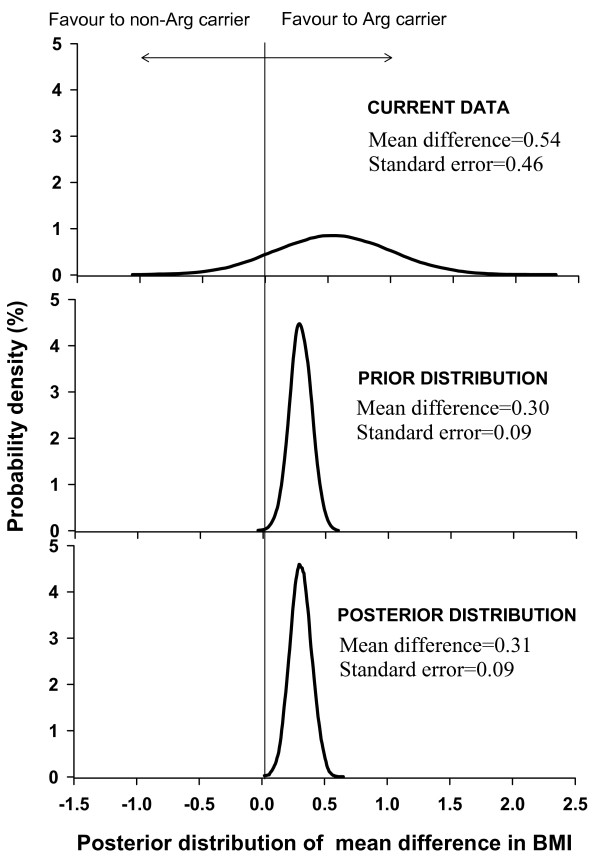
Posterior distribution of mean difference in BMI (kg/m^2^) between *Arg *carriers and non-*Arg *carriers.

In the present study, there was no significant association between ADRB3 genotypes and BMI. The overall mean difference in BMI for *Arg *carriers versus non-carriers was 0.54 kg/m^2 ^(95% CI: -0.35, 1.44; P = 0.23), or 0.13 SD. In a previous meta-analysis [26], *Arg *carriers had higher BMI than non-*Arg *carriers by 0.30 (95% CI, 0.13–0.47) kg/m^2 ^as indicated in the prior distribution (Figure 1). After updating the previous data with the present study's data, the posterior distribution showed that BMI in the *Arg *allele carriers are, on average, 0.31 kg/m^2 ^(95% CI, 0.14–0.48) higher than non-*Arg *carriers.

### ADRB3 genotypes and BMD

In multiple regression analysis of BMD measurements at both lumbar spine and femoral neck (Table 2), there were no significant associations between ADRB3 and BMD at both LS and FN sites. However, in both women and men, age and smoking history were negatively associated with femoral neck BMD, and BMI was positively associated with lumbar spine and femoral neck BMD. Although increased physical activity was not associated with BMD measured at femoral neck, it was positively associated with lumbar spine BMD. Collectively these factors (age, BMI, smoking history, calcium intake, physical activity and ADRB3 genotypes) explained about 30% and 12% of variances in femoral neck BMD in women and men respectively.

**Table 2:** ADRB3 genotype and BMD: Standardized regression coefficient from the multiple regression analyses

	LSBMD		FNBMD	
	
	Coefficient	P value	Coefficient	P value
**Women**				
Age	-0.040	0.380	-0.344	<0.01
BMI	0.366	<0.01	0.382	<0.01
Smoking	-0.041	0.37	-0.087	0.03
Calcium intake	-0.018	0.69	0.050	0.23
Physical activity	0.140	<0.01	0.035	0.40
ADRB3^a^	0.044	0.33	0.046	0.26
R^2^	0.162		0.302	

**Men**				
Age	-0.018	0.78	-0.187	<0.01
BMI	0.231	<0.01	0.240	<0.01
Smoking	-0.104	0.09	-0.149	<0.01
Calcium intake	0.091	0.14	0.048	0.01
Physical activity	0.124	0.05	0.036	0.43
ADRB3^a^	0.001	0.99	0.003	0.60
R^2^	0.074		0.120	

LSBMD, lumbar spine bone mineral density; FNBMD, femoral neck bone mineral density.

R^2^: coefficient of determination; proportion of variance of BMD (LSBMD or FNBMD) accounted for by all the factors in the model. ^a^Non-*Arg *allele carrier and *Arg *allele carrier.

### ADRB3 genotypes and fracture risk

During the follow up period, 173 women and 49 men had sustained at least one osteoporotic fracture. In women, the most common fracture site was distal radius (Colles' fracture) with an incidence rate of 24% of all the cases, followed by vertebral (23%) and hip (14%). In men, the most common fracture site was vertebrae (41%), followed by hip (16%) and ribs (14%). For both women and men, fracture individuals were older, weighed less, and had lower BMD at both femoral neck and lumbar spine compared to their non-fracture counterparts (Table 3). However, there was no significant difference between the fracture group and non-fracture group in terms of calcium intake, physical activity and smoking history.

**Table 3:** Baseline characteristics of subjects with and without fracture

	Any fracture	Non-fracture	Mean difference (95% CI)	SD^a^	P value
**Women**					
Number of subjects	173	273			
Age (y)	72 ± 8	70 ± 7	1.86 (0.46, 3.226)	0.27	<0.01
Weight (kg)	63 ± 12	66 ± 12	-2.76 (-5.14, -0.38)	-0.25	0.03
Height (cm)	159 ± 7	160 ± 6	-0.89 (-2.06, 0.27)	-0.16	0.13
BMI (kg/m^2^)	25 ± 4	26 ± 5	-0.88 (-1.76, 0)	-0.22	0.05
Ca intake (mg/day)	640 ± 379	648 ± 364	-7.70 (-78.93, 63.52)	-0.02	0.83
Physical activity (METs)	83 ± 32	79 ± 27	3.67 (-1.99, 9.32)	0.14	0.20
Femoral neck BMD (g/cm^2^)	0.72 ± 0.12	0.80 ± 0.13	-0.07 (-0.10, -0.05)	-0.63	<0.01
Lumbar spine BMD (g/cm^2^)	0.95 ± 0.17	1.04 ± 0.19	-0.09 (-0.12, -0.05)	-0.49	<0.01
Age of menopause (y)	46 ± 8	47 ± 7	-1.46 (-2.86, -0.06)	-0.14	0.04
Current/ex smoker ^b,c^	46 (26.6)	82 (30.0)			0.43

**Men**					
Number of subjects	49	216			
Age (y)	73 ± 6	69 ± 6	3.54 (1.73, 5.36)	0.67	<0.01
Weight (kg)	75 ± 11	79 ± 12	-4.05 (-7.81, -0.29)	-0.34	0.03
Height (cm)	171 ± 7	174 ± 6	-2.75 (-4.82, -0.69)	-0.48	0.01
BMI (kg/m^2^)	25 ± 3	26 ± 4	-0.54 (-1.64, 0.55)	-0.26	0.33
Ca intake (mg/day)	693 ± 475	663 ± 330	29.77 (-83.66, 143.19)	0.08	0.61
Physical activity (METs)	84 ± 53	73 ± 47	11.19 (-4.59, 26.98)	0.23	0.16
Femoral neck BMD (g/cm^2^)	0.85 ± 0.13	0.94 ± 0.15	-0.09 (-0.14, -0.05)	-0.61	<0.01
Lumbar spine BMD (g/cm^2^)	1.14 ± 0.20	1.28 ± 0.21	-0.14 (-0.21, -0.07)	-0.67	<0.01
Current/ex smoker ^b, c^	33 (67.3)	114 (52.8)			0.06

Values are mean ± SD, otherwise specified; P values are calculated using unpaired t-test, otherwise specified. BMI, body mass index; Ca, calcium; METs, metabolic equivalents;

^a ^Standardized difference; ^b^Chi-square test; ^c^N(%).

The distribution of ADRB3 genotypes in fracture and non-fracture subjects is shown in Table 4. Women with the *Arg/Arg *and *Trp/Arg *genotypes had lower incident rate of any fracture than those with the *Trp/Trp *genotype, but the difference was not statistically significant (32.8% vs. 39.7%, p = 0.30). This non-significant association was observed for all specific fracture sites. However, in men, individuals with the *Arg/Arg *and *Trp/Arg *genotypes had higher incident rate of any fracture than those with the *Trp/Trp *genotype (22.0% vs. 17.9%); the difference was also not statistically significant (p = 0.53). After adjusting for age, BMI and/or BMD, the associations between the ADRB3 polymorphism and fracture risk in women and men were not statistically significant (Table 5). There were no interaction effects between ADRB3 genotypes and age on fracture risk (data not shown).

**Table 4:** Frequency of fracture types stratified by β3-Adrenergic Receptor genotype

	Genotype		P value^a^
		
	*Trp*/*Trp*	*Arg/Arg+*	
		*Trp/Arg*	
**Women (N)**	385	61	
Non-fracture	232 (60.3)	41 (67.2)	
Any fracture	153 (39.7)	20 (32.8)	0.30
Hip fracture	37 (13.8)	4 (8.9)	0.66^b^
Vertebral fracture	53 (18.6)	5 (10.9)	0.23
Wrist/forearm	54 (18.9)	5 (10.9)	0.21

**Men (N)**	224	41	
Non-fracture	184 (82.1)	32 (78.1)	
Any fracture	40 (17.9)	9 (22.0)	0.53
Hip fracture	8 (4.2)	2 (5.9)	0.66^b^
Vertebral fracture	17 (8.5)	5 (13.5)	0.33
Wrist/forearm	3 (1.6)	1 (3.0)	0.49^b^

^a^Chi-square test, otherwise specified; ^b^Fisher's exact test

**Table 5:** Relationship between β3-Adrenergic Receptor genotypes and fracture risk

	Any fracture	Hip fracture	Vertebral fracture	Wrist/forearm^b^
**Women**				
ADRB3 unadjusted^a^	0.74 (0.42, 1.31)	0.66 (0.23,1.92)	0.56 (0.21, 1.46)	0.55 (0.21, 1.43)
Adjusted for age (1)	0.72 (0.40, 1.28)	0.66 (0.22,1.96)	0.55 (0.21, 1.43)	0.54 (0.21, 1.42)
Adjusted for FNBMD (2)	0.84 (0.46, 1.55)	0.83 (0.25, 2.77)	0.62 (0.24, 1.64)	0.61 (0.23, 1.61)
Adjusted for BMI (3)	0.76 (0.42, 1.37)	0.82 (0.27, 2.44)	0.60 (0.23, 1.56)	0.57 (0.22, 1.50)
Adjusted for (1) and (2)	0.84 (0.45, 1.55)	0.78 (0.23, 2.61)	0.60 (0.23, 1.58)	0.61 (0.23, 1.60)
Adjusted for (1) and (3)	0.74 (0.41, 1.33)	0.70 (0.23, 2.16)	0.57 (0.22, 1.51)	0.56 (0.22, 1.48)
Adjusted for (1) and (2) and (3)	0.83 (0.45, 1.53)	0.75 (0.22, 2.56)	0.60 (0.23, 1.59)	0.61 (0.23, 1.60)

**Men**				
ADRB3 unadjusted^a^	1.29 (0.57, 2.92)	1.39 (0.28, 6.76)	1.69 (0.59, 4.88)	1.84 0.19, 18.18)
Adjusted for age (1)	1.57 (0.68, 3.65)	2.87 (0.50, 16.39)	2.03 (0.69, 6.02)	-
Adjusted for FNBMD (2)	1.25 (0.52, 3.04)	1.47 (0.29, 7.46)	1.88 (0.62, 5.71)	-
Adjusted for BMI (3)	1.28 (0.56, 2.89)	1.34 (0.27, 6.58)	1.66 (0.57, 4.78)	-
Adjusted for (1) and (2)	1.47 (0.60, 3.62)	3.03 (0.52, 17.54)	2.15 (0.69, 6.67)	-
Adjusted for (1) and (3)	1.57 (0.67, 3.65)	2.85 (0.50, 16.39)	2.02 (0.68, 6.02)	-
Adjusted for (1) and (2) and (3)	1.46 (0.59, 3.62)	2.88 (0.48, 7.24)	2.15 (0.69, 6.67)	-

Values are Odds ratio and 95% CI;

^a^*Arg*-allele carriers were considered the risk factor in the model and *Trp/Trp *genotype was the reference group.

^b^adjusted OR was not calculated for wrist/forearm fracture in men due to small number of fracture.

## Discussion

Obesity is associated with increased BMD and reduced fracture risk [11,37]. Studies on polymorphisms of the ADRB3 gene and their association with BMD have been reported in the Japanese population [27,38], but not in Caucasians. The present data further suggested that there was no significant association between ADRB3 genotypes and fracture risk in a Caucasian population.

The ADRB3 genotypes have largely been investigated for association with BMI in the Japanese population, from whom a significant association has been reported. The association was characterised by individuals with the *Arg *allele having higher BMI than individuals without the *Arg *allele. In this study, individuals with the *Arg *allele also had higher BMI than individuals without the *Arg *allele, but the difference did not reach statistical significance. Nevertheless, by using the Bayesian approach (i.e., updating the current data with previous data) it was found that there was a 99% probability that BMI among those with the *Arg *allele was higher than those without the *Arg *allele.

The frequency of the *Arg *allele in the present study was similar to that reported for the Australian population [39] and other Caucasian populations [40,41], but lower than that of Asian populations [27,42-44]. To our knowledge, the present study is the first to investigate the association between ADRB3 genotypes and BMD and fracture risk in Caucasian population.

The present results suggest there was no statistically significant association between ADRB3 genotypes and BMD. This is consistent with previous findings in Japanese women [27,38] (Table 6). It has been suggested that the association between ADRB3 and BMD are more likely to be found in obese individuals than in non-obese individuals. However, in the present study, the non-significant association between ADRB3 and BMD was observed in both obese and non-obese individuals (data not shown).

**Table 6:** β3AR genotypes and bone mineral density: summary of previous and present studies

First Author and genotypes	N (%)	Femoral neck BMD (mean; SD)	Lumbar spine BMD (mean; SD)	Total body BMD (mean; SD)
Ogawa et al. [38]^a^				
*Trp*/*Trp *and *Trp*/*Arg*	267 (95.4)	NA	0.145 (1.36)	0.432 (0.93)
*Arg*/*Arg*	13 (4.6)	NA	0.179 (2.11)	-0.135 (0.93)
P-value		NA	NS	0.033
Kasumata et al. [27]^b^				
*Trp*/*Trp *and *Trp*/*Arg*	142 (97.6)	NA	NA	NA
*Arg*/*Arg*	3 (2.4)	NA	NA	NA
P-value		NS	NS	NA
The present study				
*Trp*/*Trp*	385 (86.3)	0.76 (0.13)	1.00 (0.18)	NA
*Trp*/*Arg *and *Arg*/*Arg*	61 (13.7)	0.79 (0.13)	1.04 (0.21)	NA
P-value		0.238	0.113	NA

NA, not available; NS, non-significant. ^a^Values were shown as Z-scores BMD. ^b ^Data were not shown; the authors only stated in text: "No association was demonstrated between the ER, CaSR or β3-AR genotypes and BMD, either alone or in relation to other polymorphisms".

In this current study, no significant association was observed in either sex. However, while women with the *Arg *allele appeared to have lower risk of fracture, men with the *Arg *allele had increased risk of fracture. The reason for this opposite trend was not apparent; however, stochastic fluctuation can be a contributory factor.

BMD is largely determined by genetic factors [45], and as BMI is a strong predictor of BMD [14], we thus hypothesized that ADRB3, being a candidate gene for BMI, would have pleiotropic effect on both BMD and BMI. However, the present results did not support the hypothesis, and the effect of ADRB3 seemed to be BMI specific, and the effect of ADRB3 on BMI showed a 0.31 kg/m^2 ^difference between genotypes.

The present study was based on a relatively large sample size which has been followed up for a reasonably long period of time (up to 15 years), which allows more precise delineation of effect than smaller studies. Moreover, subjects in this study are of Caucasian background, and the present results are likely to be valid in similar populations of elderly Caucasians. However, a potential limitation is that with the low prevalence of the *Arg *allele in the ADRB3 gene there are small numbers of subjects with the *Arg*/*Arg *genotype. Since fracture is a relatively rare event, the lack of fracture cases in individuals with *Arg *allele could limit the power of the study.

Furthermore, this study examined only one polymorphism which may not adequately capture the polymorphic variation within the gene, and hence limited the chance of finding an underlying association. There is also a lack of association studies investigating the relationship between ADRB3 genotypes and BMD, and no studies have investigated the association between ADRB3 genotypes and fracture risk, therefore no prior information could be drawn to reinforce our findings via the Bayesian approach.

## Conclusion

In summary, the present data suggested that in elderly men and women, there is a weak positive association between *Arg *allele of the ADRB3 gene and BMI. However, no significant associations were observed between ADRB3 polymorphisms and BMD or fracture risk, and it seems unlikely that ADRB3 has a significant predictive value for osteoporotic fracture risk.

## Abbreviations

All abbreviations are defined in the text.

## Competing interests

Dr John Eisman who serves as a consultant and receives corporate appointment from Aventis, Eli Lilly and Company, Merck Sharp & Dohme Ltd., Novartis, MPS Pharmaceuticals, Organon, Roche and Servier.

All other authors have neither financial nor non-financial competing interests that may be affected from the publication of the manuscript.

## Authors' contributions

CYW and NDN obtained and analysed data, and drafted the manuscript. NM performed the genotyping and was initially involved in the conceptual discussion of the project. JRC had an active role in the conduct of the Dubbo Osteoporosis Epidemiology Study. JAE established the Dubbo Osteoporosis Epidemiology Study. TVN had an active role in the Dubbo Osteoporosis Epidemiology Study since its inception; he was involved in the study design, data analysis, and in the conceptual discussion of the project. All authors contributed to the last version of the manuscript.

## Pre-publication history

The pre-publication history for this paper can be accessed here:


